# Dynamic functional connectivity to tile the spatiotemporal mosaic of brain states

**DOI:** 10.1162/imag_a_00364

**Published:** 2024-11-19

**Authors:** Dimitri Van De Ville, Raphaël Liégeois

**Affiliations:** Neuro-X Institute, Ecole Polytechnique Fédérale de Lausanne, Geneva, Switzerland; Department of Radiology and Medical Informatics, University of Geneva, Geneva, Switzerland; Directorate of Human and Robotic Exploration, European Astronaut Center, Cologne, Germany

**Keywords:** fMRI, functional connectivity, brain dynamics, deconvolution, null models

## Abstract

Resting-state fMRI has spurred an impressive amount of methods development, among which dynamic functional connectivity (dFC) is one important branch. However, the relevance of time-varying and time-resolved features has led to debate, to which we want to bring in our viewpoint. We argue that, while statistically many dFC features extracted from resting state are contained within a sufficiently strong null model, these features can still reflect underlying neuronal activity. The use of naturalistic experimental paradigms, at the boundary between resting state and task, is essential to validate their relevance. In parallel, leveraging methods that specifically rely on sparsity is an avenue to strengthen the statistical significance of time-resolved information carried by ongoing brain activity.

In its early days, fMRI analysis aimed at summarizing temporal information as much as possible to compensate for the low signal-to-noise ratio; that is, the celebrated general linear model (GLM) approach fits spatial parameter weight maps to 4D data using temporal regressors that are built based on the experimental paradigm. With the advent of resting-state fMRI and increased interest in spontaneous fluctuations, other approaches became necessary. In the absence of an experimental paradigm, methods characterizing spatial relationships by the use of functional connectivity (FC), a measure of statistical interdependency between pairs of time series such as Pearson’s correlation, replaced parametric mapping. Examples are seed-based functional connectivity maps and full functional connectivity matrices, which conveniently summarize an fMRI resting-state run. Blind source separation techniques such as independent components analysis (ICA) decompose resting-state fMRI data as a bilinear representation of spatial maps and associated time series. These approaches reveal spatial patterns of connectivity that are known as intrinsic functional networks as they were reminiscent of task-related activation maps, which supported their validity in resting state (Smith et al., 2009).

In the past decade, dynamic functional connectivity (dFC) took explicitly advantage of the time-varying nature of the fMRI signals and replaced the objective of summarizing the data by one of exploiting it maximally (Hutchison et al., 2013;Keilholz et al., 2017;Lurie et al., 2020;Preti et al., 2017). We emphasize that under dFC we understand any technique that allows to capture time-varying or time-resolved properties of fMRI signals, and not only extensions of classical FC. Several approaches can be distinguished based on the type of features that are considered. First, fMRI volumes can be considered directly as feature vectors. Second, fMRI volumes can be grouped in short spatiotemporal segments (Majeed et al., 2011). Third, single fMRI frames can also be “lifted” by an outer product into (instantaneous) cofluctuation patterns (Zamani Esfahlani et al., 2020). Fourth, sliding-window FC matrices can be built, which is equivalent to averaging the cofluctuation patterns with temporal windowing (Allen et al., 2014). The spatial granularity of these feature vectors can be voxels or brain regions. Subsequently, an aggregation process determines representatives of these feature vectors. The most common choice is clustering, which was explored early on in fMRI analysis (Baumgartner et al., 1998) and has known a revival with the advent of dFC, such as co-activation patterns (Liu & Duyn, 2013) and connectivity states (Allen et al., 2014). Dimensionality reduction methods such as PCA and ICA provide a latent-space representation. Temporal sequence modeling, such as hidden Markov models, goes one step further and explicitly models the succession representative feature vectors (Vidaurre et al., 2017). Those aggregated features can be regarded as brain states, that is, building blocks of ongoing activity by underlying neuronal ensembles through the spatiotemporal lens of fMRI and the action of the dFC processing choices in terms of features and aggregation.

The debate on dFC essentially boils down to if and how these states are driven by neuronal activity. Here, we bring in two perspectives into this debate.

The first issue relates to the observation that dFC excursions in real data are also present in surrogate data once the null model becomes sufficiently complex (Liégeois et al., 2021;Miller et al., 2018). An inherent problem of resting-state fMRI is that a specific sequence of brain states (i.e., from a single realization) is not meaningful when compared between different subjects and/or runs. Also, within a subject, null models such as auto-regressive models or Fourier phase randomization provide realistic BOLD fluctuations (Ladwig et al., 2022;Liégeois et al., 2021;Miller et al., 2018). In other words, the occurrence of a specific state at a specific timepoint is typically not significant. Yet, this does not prevent that such an occurrence can carry information, for instance, about arousal or attention. To draw an analogue, let us consider a human hand position that is measured over time and we are interested when grasping movements are performed. A modern robotic hand can mimic all such movements and thus traces from the human hand will not stand out against all possible robotic hand realizations, but are still meaningful for the human because they allow one to interact in a specific way with the surrounding world. Therefore, instead of whether dynamics differ from a null model, the question should rather be if the fMRI data and dFC processing steps can be considered reliable and thus do not reflect spurious fluctuations; for example, due to motion (Bolton et al., 2020;Laumann et al., 2016) or introduced by aliasing (Leonardi & Van De Ville, 2015). Therefore, it is important to validate the capability of dFC measures to capture time-resolved brain states. Since resting-state paradigms lack continuous behavioral assessment, naturalistic experimental paradigms such as movie watching (Finn & Bandettini, 2021) are ideal as they strike a balance between inter-subject synchronicity and non-trivial dynamic events, which can serve to calibrate dFC measures. Early work on inter-subject synchronization during movie-watching (Hasson et al., 2004) and impact of transient emotions on subsequent resting state (Eryilmaz et al., 2011) provided first evidence that specific brain states do occur dynamically, which has been pursued using different approaches including cofluctuation patterns (Zamani Esfahlani et al., 2020). Naturalistic stimuli generate more subtle and variable signal variations than task fMRI, and, therefore, dFC measures that do generalize well across subjects can be considered relevant for resting-state fMRI as well.

The second issue relates to which dFC measures are best suited to capture time-resolved brain states in how they reflect relevant aspects of brain activity. While typically the amplitude of the fMRI time series, after proper preprocessing steps, is considered as the input, several alternatives have been explored, for instance, reverting to the instantaneous phase extracted using the Hilbert transform of bandpass filtered time series is one other option to focus on fluctuation patterns (Cabral et al., 2017). Another approach is to identify key events in resting-state runs by exploiting the sparsity of their occurrences. To some extent, this idea was already pursued by the optimization of independent component analysis that favored non-Gaussianity and independence (Beckmann & Smith, 2004;Calhoun & Adali, 2006). But the sparsity criterion became more prominent with early work using change-point theory that modeled the fMRI time series as a piecewise-constant signal with a limited number of changes (Lindquist et al., 2007). In early work on paradigm-free mapping, sparsity-promoting hemodynamic deconvolution of the fMRI time series was used to produce an activity-inducting signal with few temporally localized and strong spikes (Gaudes et al., 2010). Finally, total activation, the temporal derivative of the deconvolved signal, was considered, resulting in the innovation signal where the spikes represent transient events that can be clustered into innovation-driven co-activation patterns (iCAPs) (Karahanoglu et al., 2013;Karahanoglu & Van De Ville, 2015). As illustrated inFigure 1, these spikes stand out much more easily against the distribution of the innovation signals from surrogate data under strong null models such as Fourier phase randomization that maintains the spatiotemporal correlation structure. The histograms of different signal representations are clearly most different between empirical and surrogate data at the level of the innovation signals, where large values are very unlikely to occur in surrogate data. Therefore, clustering using spatial patterns of innovation as feature vectors identifies state transitions at specific timepoints, from which different temporal characteristics can be derived (e.g., occurrences and dwell times). Interestingly, the temporal characteristics of conventional CAPs (Liu & Duyn, 2013;Tagliazucchi et al., 2011), which do not exploit temporal sparsity, were found identical between empirical data and surrogate data (Ladwig et al., 2022). This paradoxical difference between CAPs and iCAPs can be explained by two reasons. First, hemodynamic deconvolution brings in prior knowledge about the signal shape induced by neurovascular coupling. Second, combined with sparsity-based regularization on the deconvolved signal, one can disentangle signal originating from short events (for paradigm-free mapping) or transients (for total activation) versus general signal fluctuations. Strong surrogate data generated by Fourier phase randomization do preserve temporal and spatial smoothness of the data, but not the specific structure from sparsely driven hemodynamic responses. Along those lines, averaging operations to establish dFC measures will increase temporal and/or spatial smoothness, and thus, in virtue of the central limit theorem, bring their statistics closer to those observed for a matched Gaussian process.

**Fig. 1.**
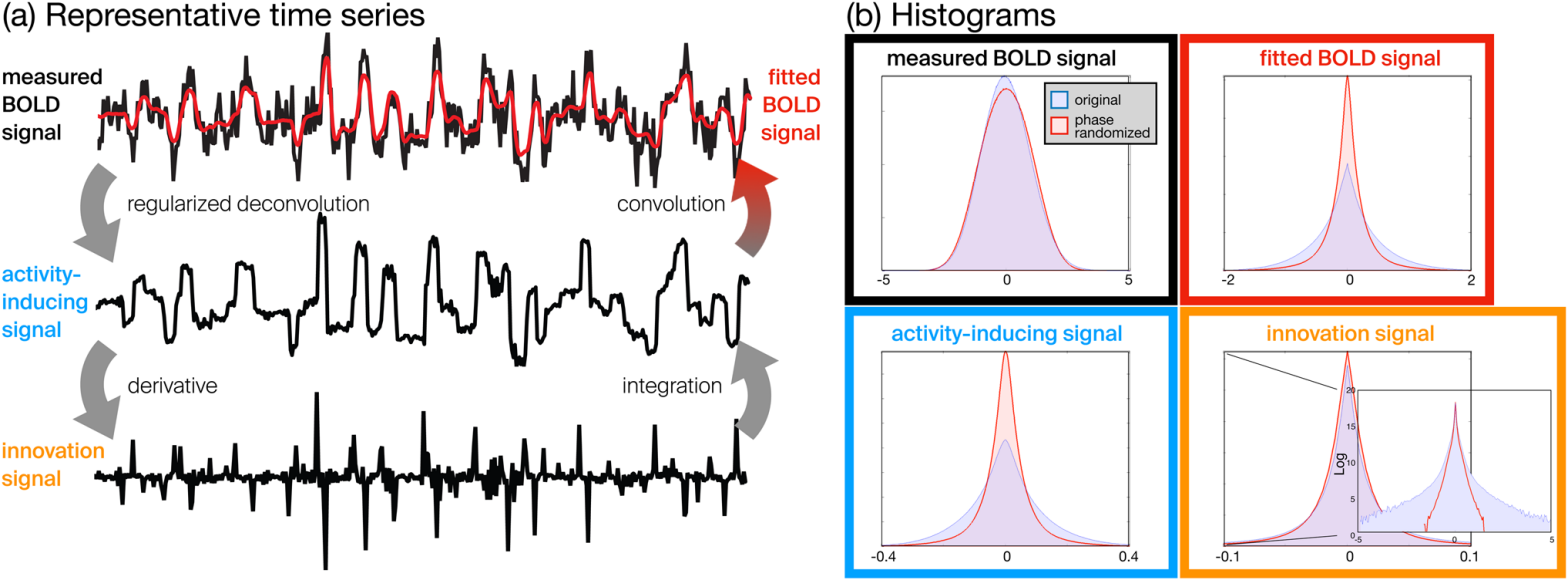
(a) Resting-state time series from posterior cingulate cortex with different representations: measured BOLD signal (black), activity-inducing signal (blue), innovation signal (orange), and fitted BOLD signal (red). The total activation framework transforms the measured time series using regularized deconvolution; that is, sparsity of the derivative of the deconvolved signal is driving the process such that peaks of the innovation signal indicate transients. (b) Histograms of the different time series illustrating how excursions of transient BOLD activity are not preserved by phase-randomized surrogate data. In particular, the heavy-tailed distribution for innovation signals indicates strong peaks that are extremely rare to be observed in surrogate data. Adapted fromKarahanoglu and Van De Ville (2015).

The attentive reader might perceive a certain contradiction between the two arguments that were developed above: The first one highlighting that beating the null model is not always meaningful, and the other one that yet some measures can do so much better than others. One should realize that null models are a moving target. Here, we considered the Fourier phase randomization technique as the gold standard to provide the most realistic surrogate data. However, given the generative model that underlies regularized hemodynamic deconvolution, one could propose surrogate data based on randomly generated piecewise-constant (thus “block type”) time series that are subsequently convolved with the hemodynamic response function. In such a case, the excursions of innovations of empirical and surrogate data would become indistinguishable again. In view of the robotic hand (the null model), the pressure profile of its fingers during grasping (a sophisticated measure) might still be significantly different from the human hand, but as technology progresses, those differences will vanish, albeit not undoing the human uniqueness.

In sum, fMRI signals reflect a rich spatiotemporal mosaic of brain activity for which dFC can provide meaningful pieces by aggregating different types of features. To support their relevance, proper validation is needed using experimental paradigms that induce key events along which dFC features align. In addition, dFC methods that leverage sudden temporal changes instead of smooth fluctuations turn out more efficient to capture these time-resolved representations in resting state.
